# Hybrid Sol–gel Coatings for Corrosion Mitigation: A Critical Review

**DOI:** 10.3390/polym12030689

**Published:** 2020-03-19

**Authors:** Rita B. Figueira

**Affiliations:** Centro de Química, Universidade do Minho, Campus de Gualtar, 4710-057 Braga, Portugal; rbacelarfigueira@quimica.uminho.pt or rita@figueira.pt

**Keywords:** sol–gel, coatings, hybrid, corrosion, metallic

## Abstract

The corrosion process is a major source of metallic material degradation, particularly in aggressive environments, such as marine ones. Corrosion progression affects the service life of a given metallic structure, which may end in structural failure, leakage, product loss and environmental pollution linked to large financial costs. According to NACE, the annual cost of corrosion worldwide was estimated, in 2016, to be around 3%–4% of the world’s gross domestic product. Therefore, the use of methodologies for corrosion mitigation are extremely important. The approaches used can be passive or active. A passive approach is preventive and may be achieved by emplacing a barrier layer, such as a coating that hinders the contact of the metallic substrate with the aggressive environment. An active approach is generally employed when the corrosion is set in. That seeks to reduce the corrosion rate when the protective barrier is already damaged and the aggressive species (i.e., corrosive agents) are in contact with the metallic substrate. In this case, this is more a remediation methodology than a preventive action, such as the use of coatings. The sol-gel synthesis process, over the past few decades, gained remarkable importance in diverse areas of application. Sol–gel allows the combination of inorganic and organic materials in a single-phase and has led to the development of organic–inorganic hybrid (OIH) coatings for several applications, including for corrosion mitigation. This manuscript succinctly reviews the fundamentals of sol–gel concepts and the parameters that influence the processing techniques. The state-of-the-art of the OIH sol–gel coatings reported in the last few years for corrosion protection, are also assessed. Lastly, a brief perspective on the limitations, standing challenges and future perspectives of the field are critically discussed.

## 1. Introduction

**Figure 1:**
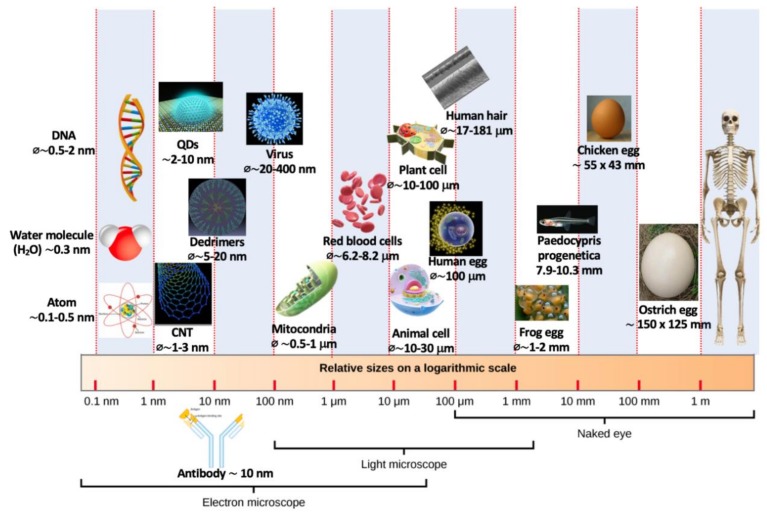
Materials scale. All the images in this figure were obtained from Wikimedia Commons. Adapted from [26].

The sol–gel synthesis process is a versatile method used to produce a wide diversity of materials, which range from inorganic glasses to complex organic–inorganic hybrid (OIH) materials [1,2,3,4,5,6,7]. Specific types of OIH materials have also been named, either as ORMOSILs (acronym created from organically modified silicates) or OMOCERs (from organically modified ceramics) depending on the precursors employed and on the synthesis route. OIH materials may be described as *multicomponent compounds that have at least one of their organic and inorganic components in the sub-micrometric and/or in the nanometric size domain* (Figure 1) [8]. The properties of the OIH materials are not the simple addition of the contribution of each individual component; instead, their properties result from the synergy created between the two components (i.e., organic and inorganic). Basically, to synthesize OIH materials through sol–gel method, it is required to introduce molecular precursors which allow the formation of the organic component. This can be achieved by [2,9,10,11,12]:

1) Adding organic precursors that are soluble in the reactional media (where hydrolysis/condensation reactions take place), although do not take part in the gel formation. The OIH material obtained by this route will display an organic component bonded to the inorganic network by van der Waals forces, or ionic or hydrogen bonds.
2) Adding organic alkoxides (R’M(OR)x), in which an organic group R’ is bonded to the element M and it is not hydrolysable. In this case, the organic and inorganic components establish covalent bonds.

The simple sol–gel processing conditions and the possibility of tuning OIH materials for specific requirements are the main reasons for their development. Moreover, OIH gel materials combine both the advantages of organic polymers (i.e., impact resistance, flexibility and light weight) and the properties of inorganic material components (i.e., chemical resistance, thermal stability and mechanical strength). Therefore, in the last few decades, a remarkable advance regarding OIH materials—controlling structure at a nanometric scale (Figure 1)—was achieved. Controlling at such a scale level allows one to obtain an OIH with certain macroscopic properties that are not present in the individual materials (organic and inorganic components). This allows one to obtain materials with distinctive properties when compared to the separated components. Therefore, OIH sol–gel materials show a huge potential for applications in a wide range of fields as diverse as optical [13], electronics [14], surface treatments [15], construction [16,17,18,19], textile [20], energy [21] and health [22], among others. The development of OIH materials, being a multidisciplinary field, involves materials engineering, chemistry, biology, physics, medicine, *etc.* Moreover, it can be considered that the development of such materials is already impacting people’s lives with significant expression in the medical field [22,23,24,25].

OIH materials, compared to pure inorganic glasses, show significant advantages, including: improvement/increase of the flexibility of the silica gel enabling one to produce thick and crack-free films; introduction of reactive functional groups within the OIH network that can be used to anchor molecular recognition groups and allow entrapping higher concentrations of species. The components employed during the synthesis will dictate the properties of the materials. For instance, the use of an inorganic network is limited in terms of implementation and new functionality input. A way to ease the shaping step and to tailor the bulk properties of the final materials is the introduction of an organic component covalently linked to the network. This can be achieved through the use of an organo-alkoxysilane. The organic component will bring flexibility to the final network modulating either the chemical or the mechanical properties [27]. Organic components can be introduced into the inorganic network as *network modifiers* or as *network formers* [2]. The *network modifiers* contribute to the functionalization of the matrix. For example, some organic precursors, such as methyltriethoxy-silane (MTES), provide flexibility [27,28], while fluoro(triethyl)silane is known for its hydrophobic properties [27,29,30]. The most used *network formers* are 3-(trimethoxysilyl)propyl-methacrylate (MEMO) [27,31], (3-aminopropyl)triethoxysilane (APTES) [32,33] and (3-glycidoxypropyl)methyltriethoxysilane (GPTMS) [27,34,35]. The mentioned precursors have to be thermally or photo-chemically cured, leading to an organic network linked to an inorganic one. It has also been reported that the addition of organic monomers to a *network former* contributes to a double reticulation and creates a winding between the organic and inorganic networks [27].

The development of OIH sol–gel coatings, based on siloxanes, for corrosion mitigation, has been widely studied in the last few years [2,31,36,37,38]. Numerous studies have been conducted since the early 1990s [39,40]. The studies performed showed that siloxanes were effective in mitigating the corrosion processes of different metallic substrates. This development was mainly due to the need for alternative environmentally friend materials and processes to replace the traditional chromium-based pre-treatments. Hexavalent chromium (Cr (VI)) is carcinogenic and shows high environmental toxicity and its use has been forbidden since 2006. However, Cr(VI) based pre-treatments improve the coating adherence to the substrate and are effective corrosion inhibitors [41,42,43] which make its replacement challenging. The use of the sol–gel method to produce OIH coatings for corrosion mitigation suits the main requirements of what should be an environmentally friendly process (i.e., excludes washing stages; it is a waste-free method) and allows one to obtain coatings with high purity and specific pore volumes and surface areas. Since it is a mild synthesis process (e.g., low temperature synthesis—often close to room temperature), the thermal volatilization and degradation of entrapped species (such as corrosion inhibitors) is reduced. As liquid precursors are used in the sol–gel process, it is possible to cast coatings in and produce thin films without the need for machining or melting [31].

Corrosion is known as one of the most severe complications in modern societies. The corrosion degradation process and subsequent rate of reaction depend on both the physical and chemical properties of the metallic substrates (e.g., steel, aluminum, titanium and respective alloys). Furthermore, the type of corrosion (e.g., pitting, bimetallic, filiform) is also determined by the metallic substrate and environment [44,45,46]. Aggressive species, such as chloride ions (Cl^−^), O_2_, and H_2_O, including the electron transport, play important roles in this process. The deterioration of the metallic substrates may lead to defects that may threaten its performance and lead to earlier failure, which is always linked to high costs, and the resulting losses each year are huge. Several cost-of-corrosion studies have been conducted by several countries (e.g., United States, United Kingdom, Japan, Australia, Kuwait, Germany, Finland, Sweden, India and China). All the studies agreed that the annual corrosion costs ranged from approximately 1% to 5% of the gross domestic product (GDP) of each nation. In 2016, a NACE study estimated a global cost of corrosion at $2.5 trillion annually, equivalent to roughly 3.4% of the global GDP [47]. Moreover, according to *360 Market Updates* [48] the global nanostructured coatings and films market was valued at million US$ in 2018, and it is expected that, considering the compound annual growth rate, this market will reach billions of US$ by the end of 2025. The main applications include oil and gas, aerospace and aviation, automotive, textiles and apparel, medical, buildings and consumer electronics industries. Therefore, several approaches are needed for corrosion mitigation. Generally, two approaches are considered; namely: a passive approach that is usually preventive and may be achieved by placing a barrier layer (e.g., coating) which hinders the contact of the metallic substrate with the aggressive environment; and an active approach that is used when the corrosion has already appeared. The damage of the protective layer allows the entrance of corrosive agents that will further attack the metallic substrate. Therefore, an active approach intends to decrease the corrosion rate and mitigate the development of further corrosion degradation of the metallic substrates. Nevertheless, it is accepted worldwide that only the combination of both methodologies can provide a reliable and durable protection of the metallic substrates against corrosion. Thus, the development of coatings that effectively prevent, mitigate and delay the corrosion process are of extreme importance. A material to be used as a protective coating must include several properties; namely, effective corrosion protection, abrasion and cracking resistance, good adhesion between the metallic surface and the coating and a long-life span. Simple OIH coatings can improve the adherence between the substrate and organic based coatings, for instance. Nevertheless, they behave only as a physical barrier. To obtain OIH with anticorrosive performance, additional functions must be introduced within the OIH network, such as corrosion inhibitors [16,49,50,51]. The alternatives proposed in the literature include OIH materials containing corrosion inhibitors such as zirconium [33,52,53,54,55,56], titanium [30,57,58] and cerium [50,55,59]. M. Catauro *et al.* synthesized OIH sol–gel materials based on zirconia (ZrO_2_) and poly(ε-caprolactone) to coat pure titanium for implants application [56]. The authors concluded that the OIH films were suitable to coat the metallic implants, to improve their bioactivity and maintain the passivation properties of the metallic substrate. Later, the same authors reported the synthesis of OIH materials by sol–gel method using TiO_2_ for the inorganic matrix and poly(ε-caprolactone) as organic component. The material produced was used to improve the performance of Ti6Al4V implants [58]. Most of the data found in the literature indicate that the OIH sol–gel materials show an enormous potential in the field of coatings for corrosion mitigation. Several multifunctional OIH coatings have been reported in the last few years showing promising results, with functions adapted according to each metallic substrate and environment [60,61,62,63,64,65,66,67,68,69,70,71,72,73,74,75,76,77].

The current review includes four main sections: the first section is dedicated to the concepts of sol–gel process from a historical perspective. The second section describes the fundamentals of the sol–gel method. The third section covers, succinctly, the main progress on the development of OIH sol–gel coatings for corrosion mitigation, including self-healing, anti-fouling and superhydrophobic functions. Finally, the fourth section outlines the challenges and prospects for future research. 

## 2. Sol–gel Process: A Historical Perspective and Applications

This section will briefly focus on the historical perspective of the first industrial interest in sol–gel materials, followed by their commercialization as coatings. A detailed and very complete historical perception on the evolution of OIH material can be found elsewhere [78]. 

**Figure 2:**
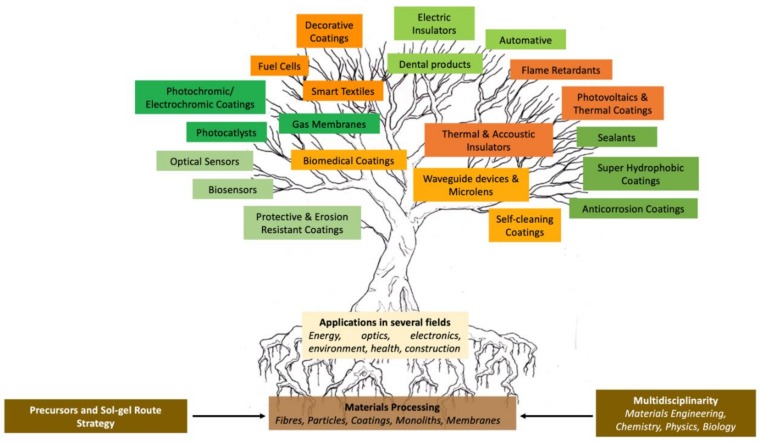
Arborescence representation of OIH materials applications. Adapted from [2].

The commercialization of sol–gel coatings appeared about a century after publication of the first scientific papers with Ebelman and Graham’s studies, in 1846, on silica gels [79]. Although noticed as early as 1846, and known to play a part in natural processes (e.g., formation of opal, glasses and ceramics), it was only during the post-World War II period that the *solution-sol–gel*, commonly known as the sol–gel process, became increasingly exploited for the preparation of glasses and other ceramic materials [80]. Graham (1861) is generally considered as being the founder of colloidal materials science [81]. However, the oldest sols prepared in a lab were made of gold colloidal particles, by Faraday in 1853 [82]. Later, in 1919, W.A. Patrick submitted a patent of a gas mask canister for vapors’ and gases’ adsorption. The innovative part of this patent was the silica-gel made of sodium silicate [83]. In the late 1930s, Geffcken and Dislich from Schott Glaswerke Company, developed the basis of the dip coating process to cover industrial glass with thin oxide layers [84]. The re-introduced attention in the sol–gel process was mainly due to the need for obtaining optical glass components (e.g., lenses, mirrors) at low temperatures without subsequent polishing. Additionally, the opportunity for modifying the materials structures at a nanoscale level (Figure 1) lead to the synthesis of advanced ceramics. From the 1940s until the end of the 1970s, the advances using the sol–gel process were mainly focused on the development of mixed OIH materials [78]. At that time, some labs, such as atomic energy in the United States of America, had already applied the sol–gel route to produce pellets and fuel powders. However, the work developed stayed secret until the 1970s [84]. From the 1980s until nowadays, a huge development in the sol–gel process was achieved. The research conducted allowed them to obtain OIH materials with applications in a wide range of areas, such as chemical and biosensors [13,85,86], in several transduction modes, from electrochemical [87,88,89,90,91] to optical [13,92,93,94,95]; biomaterials for drug delivery [24,96,97,98,99,100]; materials for improving bioactivity and biocompatibility of metallic substrates [101,102,103,104,105,106,107,108]; electronics [14]; environment [109,110,111]; optics [13,112,113]; medicine [53,114,115,116,117]; functional smart coatings [31,38,73,118]; fuel and solar cells [119,120,121,122]; and catalysts [123,124] (Figure 2). In 2018, Nourani-Vatani et al. [53] reported that a huge development for hospital equipment, and medical, dental and laboratorial devices using different metallic alloys has been achieved. However, such metallic alloys show some limitations, including low corrosion/wear resistance and unsuitable scratching and hardness properties. Therefore, to solve these drawbacks, several coatings have been proposed [57,101,105,108]. It was also concluded that zirconium-based coatings and OIH materials have been widely studied, as has the application, simultaneously, of polymers and zirconium enhanced several properties; namely, scratching, biocompatibility, bioactivity and hardness [53]. 

The sol–gel method offers several advantages when compared to traditional synthesis processes [1,36,125,126,127]; namely, the ease of fabrication and flexibility of the process, the large number of precursor reagents commercially available with tuned functional groups and the low environmental impact. Furthermore, sol–gel also allows for excellent control of the stoichiometry of the precursor; and the incorporation of different components that introduce complementary functions to the material (Figure 2), such as UV protection [128,129,130], anti-fouling [131,132], anti-reflection [133,134], moisture resistance [135], corrosion protection [15,16,31,38,136,137,138] and detection of biological components [88,139]—proteins [140] and enzymes [92,141], antibodies [142,143], DNA [95,144,145], polysaccharides [146,147], *etc.* This feature allows one to obtain, in a simple way, smart and multifunctional materials. Nowadays, the development of OIH sol–gel materials is mostly focused on green [37,49,148], safe, smart [37,149,150] and economic approaches. Ideally, these new materials should be intelligent, with properties such as self-healing abilities [73,151,152,153] or the capacity to act according with a certain environment/need or property (stimuli-responsive). OIH materials with the ability of reacting to different stimuli, such as mechanical, chemical, optical, electric and thermal ones, in order to mitigate undesired changes, is the focus of most research reported. The search for materials with the capacity to heal, adapt and behave according to each need and environment—multifunctionality—will mark the 21st century achievements.

## 3. General Concepts of the Sol–gel Process.

The sol–gel synthesis process has been widely reported in the literature [1,2,15,38,154,155,156]. Therefore, in the present manuscript, only a generic approach on the sol–gel fundamentals will be conducted.

Over the last five decades, an exhaustive development of the synthetic approaches to various oxide materials has been based on the polymerization of metal alkoxides, M(OR)_n_, namely, silicon (Si), such as tetraethoxysilane (TEOS) or tetramethoxysilane (TMOS). This process is influenced by several factors; namely: pH, temperature, catalyst, solvent, nature of alkyl group, water to alkoxide molar ratio, etc. Alkoxysilanes generally show a slow kinetic rate when compared to the titanium, aluminum or zirconium alkoxides. This allows a higher chemical control of the process, leading to materials with different properties, such as pore size and distribution, dimension and shape of the particles. Furthermore, silicon shows lower reactivity regarding chelating groups and redox process, which may lead to the formation of sub-products [157]. 

Table 1 shows chemical structures of the most-used silicon alkoxide precursors in the synthesis of OIH materials. The precursors with polymerizable functional groups, *e.g.,* epoxide (GPTMS), vinyl (VTMS) or methacrylate (MAPTS), besides allowing the formation of an organic network, also contribute to further densification of the OIH material.

The chemical reactions involved in the sol–gel process are usually divided into two steps. The first step involves the hydrolysis of metal alkoxides producing hydroxyl groups in the presence of water. The second step is followed by polycondensation of the resulting hydroxyl groups and residual alkoxyl groups, forming a three-dimensional (3D) network, establishing covalent bonds Si–O–Si in the case of silicon alkoxides. Therefore, the alkoxides and esters of inorganic acids are raw materials of utmost importance in the sol–gel technology. In the case of silicon alkoxides, the hydrolysis and condensation reactions may be catalyzed by acids or bases [158]. The structure and morphology of the final material is strongly dependent on the nature of the catalyst and pH of the reaction. The hydrolysis reactions, either catalyzed by acids or bases, are nucleophilic substitution reactions type S_N_2, characterized by a transition state in which the silicon atom is penta-coordinated. The replacement extension of the OR groups by OH groups in the silicon atoms depends on the molar ratio Si/H_2_O:

(1) $$\mathrm{Si}{\left(\mathrm{OR}\right)}_{4}+n\;H_{2}O\;\mathrm{Si}{\left(\mathrm{OR}\right)}_{4-n}{\left(\mathrm{OH}\right)}_{n}+n\;\mathrm{ROH}$$

For acidic catalysis, the following reaction takes place: 
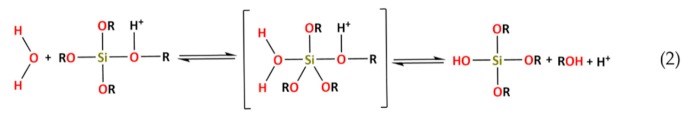


For basic catalysis, the following reaction takes place: 
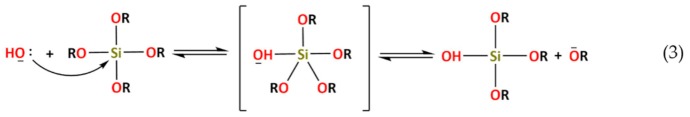


The condensation reaction of Si(OR)_4-*n*_(OH)*_n_* may also be catalyzed by acids or basis. The reactions for acidic catalysis are: 
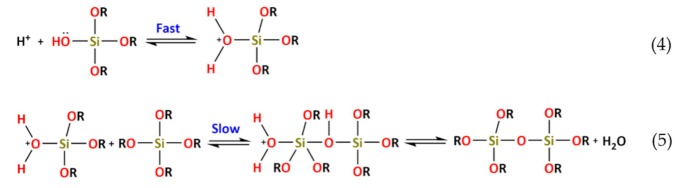


The reaction mechanism of condensation by acidic catalysis starts with the protonation of the silanol species ending with the formation of a Si–O–Si bond.

The reactions for alkaline catalysis are: 
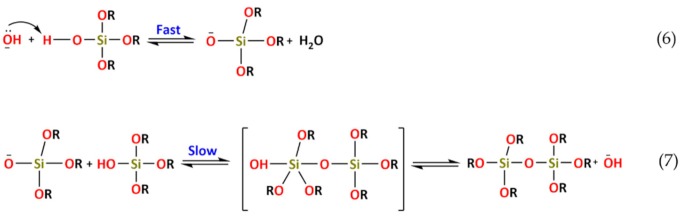


For alkaline catalysis, the specie HO^−^ removes the proton from the silanol group, forming a silanolate anion ending with the formation of a Si–O–Si bond. The reactions of alkoxysilanes, in which monomers form polymeric structures by loss of substituents, involve the formation of a colloidal suspension—*Sol*—followed by the polymerization that will lead to the formation of a 3D structure—*gel*. For acid catalysis, the alkoxysilane hydrolysis rate shows higher kinetics when compared to basic catalysis. Therefore, the acidic catalysis leads to the formation of a highly branched polymeric structure, while alkaline catalysis leads to colloidal particles with linear chains or a ringed shape. Once the gel is formed, the hybrid network needs to be dried in order to remove the water, alcohol and solvents that are formed during the hydrolysis/condensation reactions.

**Figure 3:**
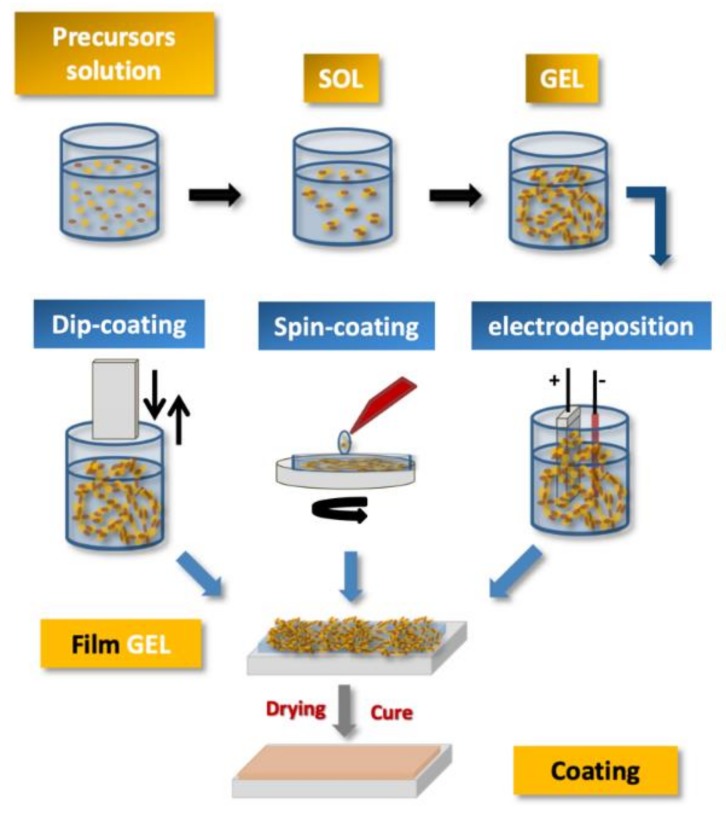
Schematic of steps and processes used to obtain sol–gel coatings [37].

The drying step has an important role, since, depending on the process used, different materials will be obtained. For instance, if the drying process takes place under normal conditions, a contraction of the gelled network occurs. The obtained dry gel, which is known as *xerogel*, may shrink between five to ten times in volume when compared to the gel before drying. The shrinkage process includes the transport of the liquid through the pores of structure and contraction of the network [1,15,38]. The *xerogel* is suitable for substrate catalysis, filters, among other applications, mainly due to low porosity and large surface area [131]. Several studies have been reported concerning the use of *xerogels* in the field of filters and membranes [159] and for carbon dioxide capture [160]. If the drying of the gel occurs in wet supercritical conditions, the shrinkage will be minimal, and the resulting product is porous and is known as an *aerogel* [1,111,161]. The material is known as a *monolith* if the dimensions of the gel are greater than scarcely millimeters [1,162,163,164]. In case of the gelation, it takes place by quick evaporation of the solvents; the materials obtained are coatings (Figure 3), films and fibers [14,154,165]. 

Nevertheless, it should be kept in mind that during the loss of volatile by-products, formed through the hydrolysis and condensation reactions, controlling the sample shrinkage during the 3D network development may be challenging and puzzling [1,6,7]. This type of material, composed by organic and inorganic components and known as OIH, is extremely attractive, since it enables an interface between two worlds of chemistry (organic and inorganic), each with unique properties that cannot be found on the individual components. 

## 4. Hybrid Sol–gel Coatings for Corrosion Mitigation 

**Figure 4:**
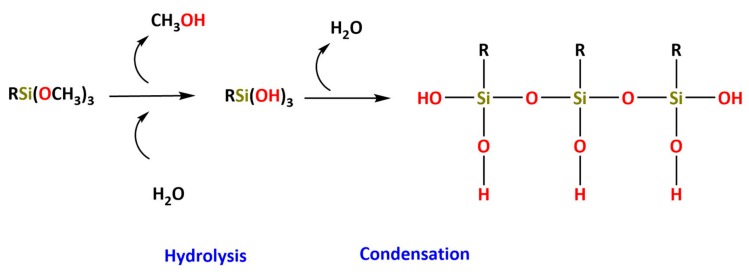
Generic two step reactions involved in the preparation of OIH materials through the sol–gel method using as the network-forming element the Si. R is typically an alkyl group. Adapted from [1].

OIHs have numerous remarkable and attractive properties with application in a wide range of fields (Figure 2). However, the huge interest involved in the development of such materials is mainly due to the convenience of the synthesis process. The sol–gel method is a flexible and simple route that allows one to obtain OIH materials in different forms (films, fibers, nanoparticles (NPs), etc.). This synthesis route is exceptionally valuable for the design and production of OIH coatings. Furthermore, sol–gel synthesis route allows one to control, strictly, the precursor stoichiometry, to modify and introduce functional groups or encapsulate different elements. Moreover, it allows one to coat substrates with large surface areas and minimum complex geometries involving simple and inexpensive equipment, as illustrated in Figure 3. It is a process that involves the ability to manipulate nanostructural (Figure 1) properties by controlling the hydrolysis, condensation reactions (Figure 4) and aggregation processes. 

The properties of OIH sol–gel materials, such as homogeneity, stability and transparency, are largely dominated by the concentrations and types of precursors, the solvent’s nature, the concentrations and types of additives (surfactants, catalysts, etc.), the pH of the reactional media, the heat treatment and the curing time [37,166]. Furthermore, the OIH gel network can be modified with a vast number of dopants [155,167,168,169,170] leading to products with singular and extraordinary properties.

The nature of each of the interactions between the organic and inorganic components, within the hybrid networks, was used to establish an OIH classification. The first classification was introduced in 1994 by Sanchez and Ribot [3] and the OIH materials were divided into two classes (class I and II). Later, in 1997, Wojcik and Klein [171] introduced a three class system. OIH materials from class I are the ones where the interactions established between the organic and inorganic components are weak (e.g., hydrogen and ionic bonds, van der Walls bonds, π−π interactions). OIH materials from class II establish strong bonds between the organic and inorganic components, such as covalent bonds. OIH class III are the ones that comprise both the type of bonds established in OIH materials from class I and II.

The requirements for a material to be used as a coating for corrosion mitigation must include effective protection against corrosion, abrasion resistance and a good adhesion between the coating and the substrate surface. The OIHs class II show properties such as high hydrophobicity, good corrosion protection, low dielectric constants or good scratch resistance [12,31]. Therefore, these chemical and physical properties make the OIH class II the ones more suitable to mitigate corrosion/oxidation of the metallic substrates. OIH class II can be easily prepared by the sol–gel process in which the alkoxysilane precursor, generally a trialkoxysilane, has an alkyl group bonded to the silicon atom producing monomers in R’Si(OR)_3_ in which R’ may be H, CH_3_, CH_2_CH_3_ or an aromatic group (Table 1). This alkyl group, by establishing a bond C-Si does not react (e.g., hydrolysis/condensation reactions) and remains covalently bonded to the silicon groups, through the C–Si bond, making part of the final material structure (Figure 4). These materials, known as polysilsesquioxane (R’SiO_1.5_) [172,173,174] have desirable properties, such as good hydrophobicity and chemical and thermal stability. The organic groups R’ are *network modifiers* and reduce the functionality of the silicon atoms, turning the silica network less brittle and rigid [175]. Another example of this type of material consists of the incorporation of a polydimethylsiloxane (PDMS) network linked to the SiO_2_ network by the co-condensation of PDMS oligomers in a sol–gel reaction [176,177]. Monomers R’Si(OR)_3_, in which R’ is an organic reactive group, such as amine, isocyanate, vinyl or epoxide (Table 1), may be used to obtain polysilsesquioxanes by sol–gel. The organic groups, through the combination of suitable monomers, may allow the formation of bridges between the silicate groups [175]. Examples of these types of products were reported by different authors [16,17,162,164]. In the last fifteen years, several review articles have been published in the design and application of OIH materials, and mostly were approached from the point of view of materials [2,3,12,27,36,112,148,178,179,180,181,182]. Table 2 shows the most relevant review manuscripts published since 2016 focused on OIH sol–gel materials. 

Table 2 shows that numerous OIH sol–gel coatings for corrosion applications were developed [30,50,137,191,192,193,194,195,196,197]. The number of reviews reported, only since 2016, clearly indicates that this field is still under development and is far from reach a matured stage. Several OIHs for corrosion mitigation have been proposed for a wide range of metallic substrates, including aluminum and its alloys [30,63,74,137,198], carbon steel [193], stainless steel [196,199,200,201] and galvanized steel [16,17,202,203,204]. Copper [205,206,207] and magnesium [208,209,210,211,212,213,214] based alloys are other types of substrates in which OIH sol–gel coatings were used. Figueira et al. [31] summarized the application of OIH (class II) siloxane-based coatings on different types of metallic substrates and respective alloys (steel, aluminum, copper, magnesium and zinc alloys) and the main precursors employed in OIH coatings synthesis. The authors showed that most of the OIH coatings developed were for aluminum-based alloys followed by iron-based alloys. Nowadays, the tendency reported for aluminum-based alloys seems to be maintained [215,216,217,218,219,220]. Moreover, in the last decade, considerable attention has been given to the development of advanced and multifunctional OIH sol–gel coating materials. Impressive advances have been reported concerning the use of carriers [49,51,149,221,222,223,224] loaded with functional abilities (e.g., self-healing [61,73,152,213], anti-fouling [132] and hydrophobicity [30,225]). 

### 4.1. Sol–gel Coatings with Self-healing Function

**Figure 5:**
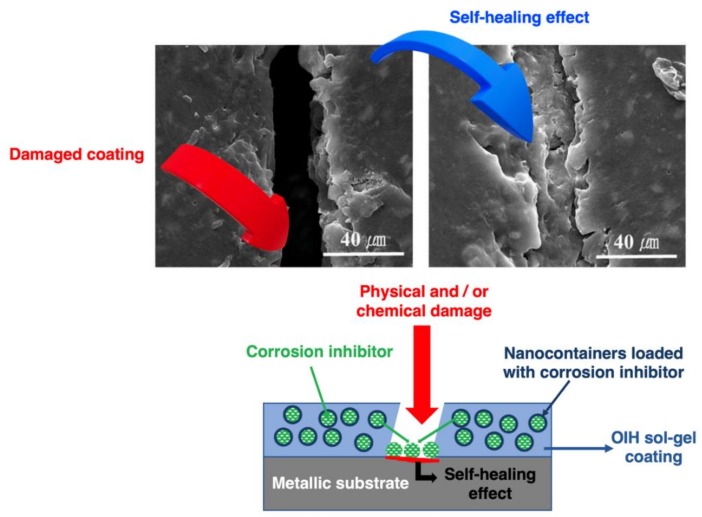
Schematic of corrosion self-healing mechanism with an OIH sol–gel coating containing nanocontainers loaded with corrosion inhibitors. Adapted from [37,233].

In the last few years, the interest in developing materials with self-healing ability led to outstanding developments [64,152,226,227,228,229,230]. Self-healing ability can be achieved at different levels of the coating system and by different strategies [231] and is known as the ability of a coating, in a certain environment, repair damaged areas due to external factors (Figure 5). According to Hia et al. [232] self-healing coatings may be classified into two types: intrinsic and extrinsic. Intrinsic coatings are the ones with a latent self-healing functionality and have the ability of heal the coatings matrices by themselves. Extrinsic coatings have a healing agent microencapsulated and embedded within the coating matrix. In this case, when the microcapsules are ruptured by damage, pH or another trigger, the healing agent is released and seals the damaged region, and the healing reaction takes place.

Strategies such as the addition of polymerizable agents [69,209] to restore/heal the damage in a polymeric coating or the inclusion of corrosion inhibitors (Figure 5) [50,153,221,234] to stop the damage spread have been reported. Metals such as cerium [50,59,200,220,235,236,237,238], lanthanum [239,240,241] or zirconium [33,53] have been incorporated in OIH sol–gel coatings in order to stop corrosion propagation [234,236,237,242,243]. Studies based on OIH coatings doped with cerium inhibitors have been reported since 2002 [235,244]. However, this review will focus only on the publications performed after 2015. Concerning the use of cerium as a corrosion inhibitor, the authors L.R.V. et al. [50] in 2017 performed a comparative study on the effect of CeO_2_ NPs and Ce(III) ions as corrosion inhibitors in silica-alumina OIH sol–gel coatings. The coatings doped with cerium nitrate (Ce(NO_3_)_3_) showed poorer corrosion behavior when compared to CeO_2_ NPs. The authors attributed this difference to the leaching out of Ce(III) ions in case of coatings doped with Ce(NO_3_)_3_. This behavior was not reported for coatings doped with CeO_2_ NPs. It was also concluded that the coatings doped with CeO_2_ NPs were more “compact,” minimizing the electrolyte ingress and therefore providing improved protection. U. Tiringer et al. [220] produced OIH sol–gel coatings, based on TEOS and GPTMS precursors and containing SiO_2_ particles and Ce(NO_3_)_3_ for corrosion mitigation of aluminum alloy AA7075 in the presence of Cl^−^. The authors concluded that the curing procedure affected the coating properties obtaining coatings with lower roughness, reduced thickness and higher density by using a curing heating ramp. The incorporation of Ce(NO_3_)_3_ increased the roughness and thickness linked to a network with higher porosity. The authors showed that the porosity allowed the release and migration of the incorporated Ce(III) ions. The electrochemical data indicated that the coatings doped with Ce(NO_3_)_3_ and cured with heating ramp exhibited improved corrosion properties. Later, in 2019 the same authors [238], reported the synthesis of OIH sol–gel coatings based on the same precursors also doped and undoped with Ce(NO_3_)_3_. The effects of curing temperature and the addition of Ce(NO_3_)_3_ on the degree of polycondensation of the OIH sols were studied. It was concluded that the addition of Ce(NO_3_)_3_ did not affect the viscosity of sols and improved their thermal stability. Furthermore, the authors revealed that the addition of Ce(NO_3_)_3_ acted as an initiator, stimulating the polycondensation reaction at lower temperatures. Earlier this year [59], T.T. Thai, et al. developed OIH sol–gel coatings doped with Ce (III) ions by using montmorillonite as container. The electrochemical studies showed that the coatings were effective at protecting the metallic substrate. At a low concentration of cerium-modified clay particles (0.5% wt.), the Ce (III) played a role as effective cathodic inhibitor of the metallic substrate. The leached cerium rate obtained was about 70%, and the authors showed that it was from the Ce(III) ions that were deposited, in the beginning, on the external layer of the clay platelets. In the end, it was concluded that the Ce(III) ions at the external layer of clay platelets induced the formation of Ce(OH)_2_, strengthening the corrosion protection of the OIH coating [59].

In the case of using lanthanum as a corrosion inhibitor, P. Balan et al. [240] in 2016 reported a study on the electrochemical behavior of modified silane films with lanthanum triflate salt (LTS) and/or silica NPs. The incorporation of LTS or silica NPs enhanced the anti-corrosion behavior of the silane films when compared to pure films. The films modified by both LTS and silica NPs showed improved corrosion protection. The authors also concluded that the direct addition LTS could compromise the coating barrier properties and the stability of the silane solution. These limitations were improved by incorporating La^3+^ cations in amorphous silica NPs before being embedded into the silane film. Two years later, J. Peña-Poza et al. [241] studied TEOS sol–gel based coatings doped with lanthanum acetate/nitrate on different metallic substrates (i.e., copper, bronze, lead and steel). The authors concluded that La_2_O_3_ was adequate to incorporate La^3+^ ions within the silica matrix. Furthermore, the produced coatings mitigated the corrosion process on copper, bronze and lead, while steel substrates required thicker coatings.

Ideally, the addition of species (e.g., corrosion inhibitors and polymerizable species) to improve the OIH coating’s performance must be incorporated within the coating matrix and released when required, i.e., when corrosion the process initiates, in order to delay further corrosion development. It has been reported in different studies that the direct incorporation of these species may interfere with the curing of the coating material and compromise its physico-chemical performances [16,203,245]. Therefore, the selection of suitable carriers/nanocontainers, to ensure their release in response to a trigger (stimulus-responsive), is of extreme importance [67,151,228]. The trigger which can be physical or chemical (i.e., crack propagation, the presence of moisture, pH, aggressive species) [227,235,246,247,248,249,250]. Furthermore, these nanocontainers must be able to stock the required species in sufficient amounts and be compatible with the coating itself. The most commonly used reservoirs reported in the literature include urea-formaldehyde capsules [246,247,248,249], hydroxyapatite particles [57,251], chitosan [243,252] and halloysite nanotubes [213,221]. Several OIH sol–gel coatings have been reported as smart with self-healing abilities, such as the remarkable works reported by the M.G.S. Ferreira group [63,76,149,150,253,254,255,256,257,258] and Adsul et al. [66,213,259].

Adsul et al. [66,213] studied the effect of loading cationic corrosion inhibitors (Ce^3+^/Zr^4+^) into halloysite nanotubes, which were afterwards incorporated within an OIH sol–gel matrix and deposited on magnesium alloy substrates. The authors concluded the Ce^3+^/Zr^4+^ loaded halloysite nanotubes dispersed within the OIH coating provided suitable corrosion protection of the substrate when exposed to 3.5% NaCl. The healing properties were confirmed through weight loss and electrochemical techniques. The same research group [259] also studied the incorporation of the same corrosion inhibitors into aluminum pillared montmorillonite clay. The montmorillonite clay was dispersed as received within the matrix sol–gel coatings. The coatings mitigated the corrosion process during the beginning of exposure to aggressive environment. Another coating in which the pillared clay was modified, by evacuation of a mixture of corrosion inhibitor and pillared montmorillonite, provided improved corrosion protection for higher exposure periods in the same environment. This was shown by electrochemical and weight loss measurements. However, by increasing the coating exposure time in the aggressive environment, the coating suffered deterioration. It was also proven that the coatings provided autonomous healing in case of long-time exposure. 

The self-healing mechanism in corrosion mitigation may be assessed and validated by several electrochemical tools. The most common techniques reported to assess the self-healing and corrosion inhibition of such materials are scanning vibrating electrode technique (SVET), scanning ion selective electrode technique (SIET) and localized impedance spectroscopy (LEIS) [231]. 

### 4.2. Sol–gel Coatings with Anti-Fouling Function

Anti-fouling is another important function to enhance the corrosion protection of a coating. Coatings designed for this purpose are generally based on dense organic matrices, containing wide-spectrum biocides such as tributyltin and tributyltin oxide. These biocides are one of the major contaminants in marine and freshwater ecosystems [260], and nowadays their use is forbidden. Therefore, there is an emergent interest in develop new coatings with controlled release of benign biocides [141,261,262,263,264,265,266]. The use of nanocontainers loaded with biocides is considered a very attractive methodology, since a controlled release can minimize the environmental impact. 

In the last 20 years, anti-fouling coatings have been extensively studied [110,141,261,267,268,269,270,271,272,273,274], including sol–gel coatings with anti-fouling properties [131,132,262,275]. Although many approaches and routes have been proposed [131,132,231,262,263,264,276,277,278,279], there is still an absence of long-term and effective solutions. Generally, two corrosion mechanisms in the marine environment are considered; i.e., microbiologically influenced corrosion (MIC) and marine bio-fouling. Y. Li and C. Ning [280] published a review focused on the research progress regarding these two mechanisms. The authors concluded that MIC and bio-fouling are prevented by the control of the microorganisms’ activities in biofilms, their adhesion and their formation of biofilms. It was also concluded that the performance of antifouling coatings has been improved by the modification of self-polishing copolymers and the development of degradable polymers. Conducting polymers were also developed and introduced to mitigate corrosion and biofouling. 

A wide range of encapsulated agents for fouling mitigation were proposed and assessed, including enzymes [88,141,281,282,283], ZnO_2_ NPs [284], silver and benzalkonium chloride [265], zinc pyrithione [264] and chlorhexidine [275]. The encapsulation of enzymes showed auspicious results [141,281,282,283]. For example, M. Meißler, et al. [283] reported the combination of a titanium binding peptide to a linear peptide-block-poly(ethylene glycol). The adsorption function of the peptide segments within peptide-PEG conjugates was weakened by enzymatic proteolysis. It was concluded that the adsorption properties related to the titanium substrate were fully suppressed, showing potential for antifouling surfaces in the biomedical field. Coatings combining both anti-fouling and anti-corrosion functions have also been reported [265,285,286,287]. Arukalam et al., for instance, prepared perfluorodecyltrichlorosilane-based poly(dimethylsiloxane)-ZnO coatings for fouling and corrosion mitigation. The authors chose the ZnO NPs to study the influences of the particles’ nature: the hydrophilic and anti-fouling properties. The efficacy of the produced coatings was assessed, and it was concluded that they were promising for such application. E. Wallström et al. [264] reported an auspicious route to introduce anti-fouling functionalities within a coating, wherein it was shown that the use of zinc pyrithione reduced the release rate of the biocide when compared to the release rate for the free biocide particles in demineralised water. The authors also showed that the water uptake in the paint had a major impact on the biocide release and was dependent on a number of properties, such as the binder system, pigmentation, the amount of gel and the active component included. In the end, the authors concluded that to produce formulations without the use of bio-accumulative compounds and to minimize the biocide component, it was necessary to take into account gel composition, paint formulation and water exposure. Oldani et al. [132] in 2016, reported a study where the use of OIH sol–gel coatings was promising for producing coatings for fouling mitigation. The authors combined a commercial a,v-substituted perfluoropolyether with TEOS for deposition on stainless steel substrates. It was concluded that to obtain a suitable balance between the hydrophobic properties and the film formation, the amount of polymer ranged between 70 and 80 wt.%. The OIH coatings reported showed a water contact angle (WCA) between 130° and 140° and good chemical resistance. M. Barletta et al. [261] developed a promising and effective “biocide-free” antifouling coating system. The proposed system involved the use of a polyester-modified, OH-rich polysiloxane that was cured through reactions with different commercially isocyanate hardeners. The hydrophilic and hydrophobic domains were introduced within the system on the curing agent using PEG-ilate alkyl and cyclohexyl substituents, respectively. It was concluded that all the samples showed improved results when compared to samples coated with a traditional paint. S. Holberg et al. [285] recently reported the synthesis of a hydrophilic, biocide-free fouling-release coating. The authors prepared coatings from commercial precursors by dispersing a polydimethyl siloxane (silicone,PDMS)-polyethyleneglycol triblock-copolymer in PDMS (PEG-PDMS-PEG). It was shown that the fouling growth in a *Pseudomonas aeruginosa* culture test was reduced compared to PDMS without PEG or to steel.

### 4.3. Coatings with Superhydrophobic Function

Micro- and nanopores in the coatings may lead to the entrance of water and aggressive species, causing coating blistering; then peeling; and subsequently, corrosion of the metallic substrates. Therefore, an interesting approach to prevent corrosion is the development of superhydrophobic surfaces. A coating shows hydrophobic properties when the WCA on a surface is above 90°. In the case of superhydrophobic coatings, they require a WCA above 150° and a contact angle hysteresis (CAH) lower than 10° [288,289]. CAH is expressed as *the difference between the advancing and the receding WCA of a water droplet that expands or shrinks on the surface* [290]. Additionally, these coatings should show a roll-off angle lower than 10°. According to K.K. Varanasi et al., the roll-off angle is defined as the *lowest angle that a surface needs to be inclined at before the water droplet slide or rolls off it* [291]. The reduction of the WCA and contact time on the superhydrophobic surfaces mitigate the interaction between the metallic substrates and the aqueous corrosive species. This behavior allows to enhance the corrosion protection of the substrates by indirectly avoiding the contact between the metallic surface and the aggressive species [292,293,294,295]. In the last few years, the literature reported on this type of coatings increased notably [60,291,292,293,296,297,298,299]. Numerous techniques and methods have been used in the development of superhydrophobic surfaces [300,301,302], and the sol–gel process, as the versatile method that it is, was no exception [294,299,303,304,305,306,307]. Recently, a very detailed and comprehensive review, focused on the latest developments of superhydrophobic coatings for corrosion mitigation, has been published [308]. The challenges and limitations of the synthesis of stable superhydrophobic coatings were also included and discussed. One of the main advantages of superhydrophobic coatings is that these types of materials may include other properties, such as anti-fouling [276], anti-icing [309] and bio-corrosion [231]. According to a review published by Montemor [231] in 2014, several reported encapsulation-based strategies seem to be promising; however, only a few studies were reported which validated the proposed strategies regarding the corrosion protection behavior [310,311,312]. However, important advances and developments on superhydrophobic surfaces have been achieved in the last five years [60,132,136,217,225,240,292,294,297,300,313].

OIH sol–gel coatings with superhydrophobic properties for corrosion mitigation have been reported for different substrates, such as aluminum [295,311,314,315,316,317,318], copper [225,319], magnesium [320], mild steel [321,322] and stainless steel [323]. Generally, OIH sol–gel coatings show improved UV and thermal stability when compared to organic coatings due to the high strength of the Si–O bond. Most of the developments were for application in aluminum substrates. Lee and Hwang [314] reported the development of a superhydrophobic surface for Al/Si alloy by sol–gel process. The authors fabricated a silica gel layer from the Si content of the alloy itself without the need for treatment with high temperatures with an average WCA of 166.04°. By removing one step (temperature treatment), the fabrication of such coatings becomes easier, lower in cost and enabling of its application in industry. The superhydrophobic properties can be obtained by different encapsulation routes. Wang et al. reported the storage of Ca(OH)_2_ inside microcapsules and concluded that these show appropriate superhydrophobic performances, including regenerative ability [324]. Porous silica capsules were also reported and tested by several authors [305,310,325,326,327,328]. These proved to be efficient in the development of superhydrophobic surfaces for diverse applications. Caldarelli et al. [225] reported the synthesis of a OIH superhydrophobic sol–gel coating for copper metallic substrates. The sandblasted copper substrates were coated with an alcoholic alumina sol followed by thermal annealing that was coated afterwards with a fluoroalkylsilane solution. The two-step coating deposition was performed using dip-coating technique. The authors obtained a superhydrophobic coating, and for a WCA of 180° the thermal treatment should be between 200 °C and 250 °C. Zhang et al. [322], in 2016, produced superhydrophobic films to coat mild steel substrates. The authors obtained films by using a two-step route that included the electrodeposition of silica film followed by modification with a long alkyl-chained organic silane (dodecyltrimethoxysilane). It was concluded that the produced coatings showed improved corrosion resistance compared to conventional dodecyltrimethoxysilane-silanized coatings in aggressive environments. 

A. Fihri et al. [188] published a review paper where the superhydrophobic coatings reported in the literature, until 2017, for steel protection, and their performances, were debated. The diverse models used to assess the wettability of a surface, and the approaches used to produce superhydrophobic coatings and their impacts on the corrosion mitigation of steel, were also revised. The authors concluded that remarkable advances in the development of superhydrophobic coatings have been achieved during the last two decades. However, most of the advances reported in the literature for the fabrication of such coatings still face many challenges for large-scale deployment. The main challenges identified were the short service life; low curing temperature and adhesion performance; low mechanical robustness; and the high costs involved. Therefore, the authors believe that due to the constraints still present in the development of such coatings, the research will progress towards overcoming these drawbacks in this field.

Superhydrophobicity, besides improving anti-fouling function, allows the encapsulation of active agents, as mentioned earlier. Therefore, the surface finishing can deliver both functionalities [231]. The sol–gel method and the use of silica particles are easy-going routes for achieving anti-fouling, anticorrosion and superhydrophobic coatings. 

## 5. Challenges and Prospects for the Future Research 

Nowadays, many of the effective coatings for corrosion protection represent dangers to the environment. Therefore, the key is the development of innovative materials with improved performances and promoting a sustainable environment at the same time. 

The corrosion mitigation/prevention of metallic substrates is not only an engineering effort, but also a fundamental scientific difficulty. Ideally, an anticorrosion coating should be designed to be stimulus-responsive, multifunctional, resistant and durable in a wide range of applications. Coatings for corrosion mitigation are normally exposed to complex environments. Therefore, studies on how the different aggressive species (i.e., Cl^−^) and water behave and react within the coating at atomic and nanoscales are needed. 

The opportunity of hosting different functionalities broadens the range of applications for OIH sol–gel materials, which can be functionalized with carriers loaded with different agents. However, the development of this type of material requires multidisciplinary collaboration, including materials science, biology and polymer chemistry. Ideas for the encapsulation of anti-fouling species have been transferred from the biological and medical fields. The OIH sol–gel materials’ features allow one to obtain multifunctional coatings, such as self-responsive materials with anti-fouling, anticorrosion and superhydrophobic functions within the same matrix. The existent literature clearly shows that the distinctive properties of the OIH sol–gel materials can be successfully employed for the development of coatings for corrosion mitigation of different metallic surfaces in challenging environments. However, further studies should be performed using different corrosion inhibitors and different trigger stimuli. Stability and long-term healing capacity research should also be considered in depth, since these agents should maintain their function abilities for an extended period (i.e. at least 20 years). Additionally, multi-damaging and the size of the area damaged may be a drawback for these coatings and should be further investigated. On the other hand, sustainability at a large scale, toxicity and the effects of inherent properties of OIH coatings remain only vaguely explored. Although several OIH coatings for corrosion mitigation have been subjected to intensive research, it seems that further investigations are still necessary for both fundamental and applied aspects. The synthesis of OIH multifunctional coatings (e.g., anticorrosion properties, high hydrophobicity, suitable adhesion performance, self-healing abilities) with extended service lives—highly required in order to develop commercial scale products—has not been accomplished yet.

Smart coatings with self-healing functions and stimulus-responsiveness are potentially the future of developments. Self-heling by the incorporation of responsive additives within the OIH coating materials enables, due to external stimulus (e.g., pH, the presence of Cl^−^, UV, temperature and/or humidity), the restoration of the material structure and recover the integrity of the protective coating. Hence, suppressing the beginning of corrosion process and increasing the service life of the metallic materials. Additionally, the incorporation of inhibitors that can mitigate and control the corrosive substances ingress will provide extra protection together with the coating structure restored. This direction in the development of smart coatings for corrosion mitigation will for certain mark the 21st century achievements.

## Figures and Tables

**Table 1:** Abbreviation, chemical name and structure of each of the alkoxysilanes most used as precursors for synthesis of OIH materials.

Chemical Name	Abbreviation	Chemical Structures
Tetraethoxysilane	TEOS	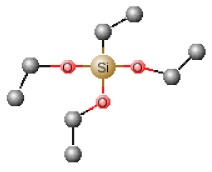
Tetramethylorthosilicate	TMOS	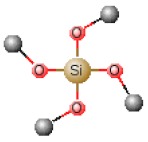
Methyltriethoxysilane	MTES	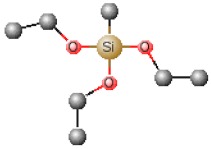
Methyltrimethoxysilane	MTMS	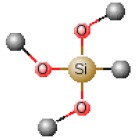
Vinyltrimethoxysilane	VTMS	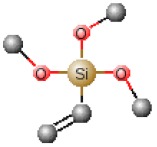
Phenyltrimethoxysilane	PTMS	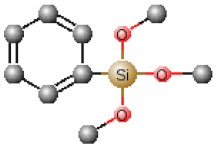
3-Aminopropyltrimethoxysilane	APTMS	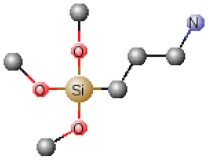
Aminopropyltriethoxysilane	APTES	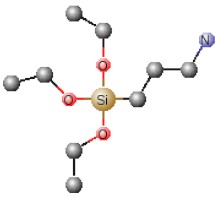
N-(2-aminoethyl) 3-aminopropyltrimethoxysilane	AEAPS	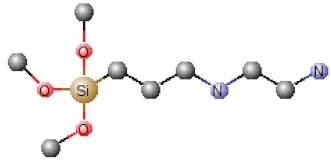
3 - Glycidoxypropyltrimethoxysilane	GPTMS	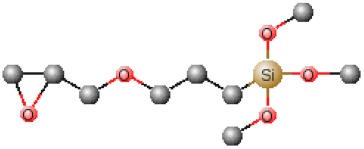
3-Methacryloxypropyltrimethoxysilane	MAPTS	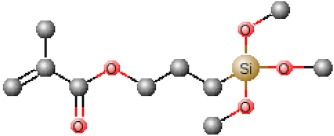

**Table 2:** The most relevant review manuscripts focused on OIH sol–gel materials published in the last few years.

Year	Reference	Discussed subject matter
2016	[183]	Principles and morphologies of microcapsules, purpose of microencapsulation,physical, and chemical techniques and healing mechanisms for the coating industry were debated.
	[184]	The progress made in the synthesis of several sol–gel-derived materials was reviewed. The main achievements in the field of anticorrosion coatings were also debated.
	[37]	Summary of the main research achievements in the development, of OIH sol–gel coatings for corrosion mitigation of steel and aluminum substrates.
	[185]	The general features of OIH coatings, and recent developments were summarized.
	[137]	Summary of the latest achievements and strategies for the sol–gel process parameters and other factors that influence the corrosion properties of the OIH coatings for corrosion protection of aluminum-based alloys.
	[20]	Overview of sol–gel technology where the fabric functions that can be achieved by this technology are debated including anticorrosion coatings.
2017	[186]	Advances in smart coatings, including OIH coatings, with response to different stimuli and damage modes were reviewed. Emphasis was on corrosion sensing, self-cleaning, anti-fouling, and self-healing polymeric coating systems.
	[187]	Recent applications using PDMS polymers for anticorrosion, anti-biofouling, anti-icing, flame-resistant and self-cleaning, anti-reflection were reviewed.
	[188]	Overview of the superhydrophobic coatings (including OIH sol–gel) reported in literature for steel protection and their performances.
2018	[138]	Different solutions for slow down the corrosion processes of metallic substrates by using the oxides and doped oxides obtained by the sol–gel method were discussed.
	[189]	Analysis of some of the most representative examples of the application of electrochemical techniques such as EIS, PDP; SVET, SIET, SKP and LEIS to determine the exact mechanism of protection offered by sol–gel coatings on metallic substrates.
	[78]	Description of the historical perception of OIH material science. The major periods linked to the genesis of OIH materials were discussed.
2019	[190]	The main aspects of the use of silicon polymers for coatings were debated. The advantages and disadvantages of these materials, and the processing methods developed were discussed.

## Abbreviations

3-D	Three-dimensional
EIS	Electrochemical impedance spectroscopy
CAH	Contact angle hysteresis
CNT	Carbon nanotubes
DNA	Deoxyribonucleic acid
GDP	Gross domestic product
LEIS	Localized electrochemical impedance spectroscopy
LTS	Lanthanum triflate salt
MIC	Microbiologically influenced corrosion
MTES	Methyltriethoxy-silane
NPs	Nanoparticles
OIH	Organic-inorganic hybrid
PDP	Potentiodynamic polarization
QDs	Quantum dots
SIET	Scanning ion-selective electrode technique
SVET	Scanning vibrating electrode technique
SKP	Scanning Kelvin probe
TEOS	Tetraethoxysilane
WCA	Water contact angle
